# Improvement of LV functional performance in the chronic total coronary occlusion during the late stage is associated with the extensive collateral development

**DOI:** 10.1186/1532-429X-13-S1-O52

**Published:** 2011-02-02

**Authors:** Yuesong Yang, Bradley Strauss, Beiping Qiang, Azriel Osherov, John J Graham, Garry Liu, Xiuling Qi, Nigel R Munce, Michelle Ladouceur-Wodzk, Normand Robert, Alexander J Dick, Graham A Wright

**Affiliations:** 1 Sunnybrook Health Sciences Centre, Toronto, ON, Canada; 2 St.Michael Hospital, Toronto, ON, Canada; 3 Ottawa Heart Institute, Toronto, ON, Canada

## Purpose

To investigate whether left ventricular (LV) function and regional wall motion improvement are associated with the extent of collateral development in coronary chronic total occlusion (CTO).

## Methods

In nine pigs a CTO was created by percutaneously inserting a fibrin plug (AngiosealΤΜ) into the mid-to-distal left anterior descending artery (LAD). Animals were studied six (n=3) or twelve weeks (n=6) later prior to sacrifice. An x-ray angiogram confirmed the LAD CTO at those time points. Cardiac MR (CMR) studies were then conducted on a 1.5T (n=6) or on a 3.0T MRI system (n=3), which included SSFP short axis sections for wall motion and post-gadolinium LGE-MRIs for viability. After sacrifice, both right and left coronary systems were injected with Microfil. X-ray or MSCT angiography of the fixed heart was obtained. A longitudinal cardiac section including the CTO lesion, proximal/distal LAD and the borders of infarction was removed and prepared in gel, then imaged in a micro-CT system at 27 micron resolution. LV functional parameters including wall thickness at end-systole (WTES) and end-diastole (WTED) were measured in border zone, infarct and remote region. Systolic wall thickening (SWT) was calculated using as (WTES-WTED) x100/WTED. CMR and micro-CT data were processed using commercial software. The extent of collaterals on micro-CT images was rated qualitatively using a score from 0 to 3, indicating that no, minimal, moderate, or extensive collaterals were observed. A Student’s t-test was used for the statistical significance of differences between measurements at 6 and 12 weeks.

## Results

**Figure 1:**
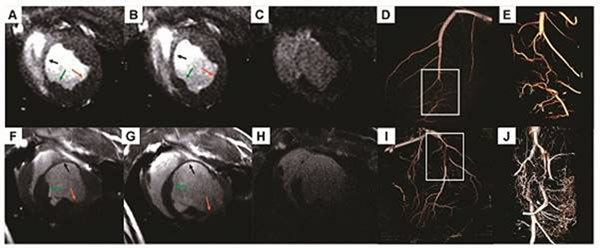
Compared to the minimal collateral formation in the 6 weeks’ CTO animal (A-E), extensive collateral development was observed in the 12 weeks’ CTO animal (F-J), which is consistent with the increased systolic wall thickening and better LV function performance measured from cine SSFP images in the late stage of CTO. A, F: end-systolic SSFP images; B, G: end-diastolic SSFP images; C, H: LGE-MRI images; D, I: rotational x-ray angiogram; E, J: 3D micro-CT images reconstructed from highlighted box regions of D and I. Black, green and orange arrows indicating infarct, border and remote region.

LGE-MRI determined the presence of LV myocardial infarction (MI). Tables 1 and 2 summarize the results of global and regional LV function measurements at both time points. Ejection fraction (LVEF) at 12 weeks was significantly greater than at 6 weeks (39.45±5.38% vs. 26.27±5.77%, P=0.01) although the extent of infarct was similar between these two groups (P=0.16). In border zone the WTES (11.31±1.72 vs. 8.67±0.57mm, P=0.04) and SWT (68.31±11.55% vs. 35.87±19.14%, P=0.01) increased at 12 versus 6 weeks. On the visual scores of collateral development, between 6 and 12 weeks, there was an increase in collateral number (1.33±0.58 vs. 2.83±0.41, p<0.003). Figure 1 similarly illustrates increased collateral development at the later time point.

**Table 1:** KV function measurements in coronary CTO pigs

CTO duration	LVEF (%)	EDV (ml)	ESV (ml)	SV (ml)	CO (L)	LVM (g)	LV-MI (g)	% (M/LVM)
6 weeks (n=3, mean±SD)	26.27 ± 5.77	89.64 ± 20.59	65.73 ± 14/47	23.90 ± 8.33	1.61 ± 0.67	71.63 ± 15.39	7.77 ± 2.75	11.45 ± 5.67
12 weeks (n=6, mean±SD)	39.45 ± 5.38	185.90 ± 13.16	113.38 ± 14.51	73.60 ± 9.60	5.71 ± 1.21	129.11 ± 10.06	10.56 ± 2.44	8.21 ± 1.96
P value	0.0115	0.0001	0.0324	0.0001	0.0011	0.0002	0.1637	0.2351

Note: CTO-Chronic total occlusion, LVEF-Left ventricular ejection fraction, EDV-End-diastolic volume, ESV-End-systolic volume, SV-Stroke volume, CO-Cardiac output, LVM-Left ventricular mass at end-diastolic phase, LV-MI-Myocardial infarction mass.

**Table 2:** Regional LV wall thickness and systolic wall thickening in coronary CTO pigs

CTO duration	B-WTES (mm)	B-WTED (mm)	B-Wall thickening	I-WTES (mm)	I-WTED (mm)	R-WTES (mm)	R-WTED (mm)	R-Wall thickening (%)
6 weeks (n=3, mean±SD)	8.67 ± 0.57	6.51 ± 1.36	35.87 ± 19.14	3.19 ± 0.49	2.82 ± 0.45	13.54 ± 2.16	8.38 ± 1.55	62.1 ± 5.10
12 weeks (n=6, mean±SD)	11.31 ± 1.72	6.78 ± 1.31	68.31 ± 11.55	2.43 ± 0.64	2.07 ± 1.02	13.89 ± 3.22	8.72 ± 1.74	58.69 ± 9.58
P value	0.0403	0.7834	0.0142	0.1186	0.2756	0.8710	0.7828	0.5881

Note: CTO-Chronic total occlusion, B-Border zone, I-Infarction segment, R-Remote region, WTES-End-systolic wall thickness, WTED-End-diastolic wall thickness.

## Conclusions

Extensive collateral development during the late stage of myocardial repair after CTO may contribute to LV functional improvement through increased SWT in the border zone. This provides a potential explanation for preserved LV function witnessed in some patients with CTO.

